# Hemopexin alleviates sterile inflammation in ischemia-reperfusion-induced lung injury

**DOI:** 10.3389/fimmu.2024.1451577

**Published:** 2024-10-04

**Authors:** Tomoyuki Nakagiri, Nadine R. Köhler, Sabina Janciauskiene, Lavinia Neubert, Ann-Kathrin Knöfel, Pooja Pradhan, Arjang Ruhparwar, Fabio Ius, Stephan Immenschuh

**Affiliations:** ^1^ Department of Cardiothoracic, Transplantation, and Vascular Surgery, Hannover Medical School, Hannover, Germany; ^2^ Biomedical Research in Endstage and Obstructive Lung Disease Hannover (BREATH), German Center for Lung Research (DZL), Hannover, Germany; ^3^ Department of Respiratory Medicine, Hannover Medical School, Hannover, Germany; ^4^ Department of Genetics and Clinical Immunology, The Institute of Tuberculosis and Lung Diseases, Warsaw, Poland; ^5^ Institute of Pathology, Hannover Medical School, Hannover, Germany; ^6^ Institute of Transfusion Medicine and Transplant Engineering, Hannover Medical School, Hannover, Germany

**Keywords:** heme, hemopexin, ischemia-reperfusion injury, lung, sterile inflammation

## Abstract

**Introduction:**

Pulmonary ischemia-reperfusion (IR) injury (IRI) plays a significant role in various lung disorders and is a key factor in the development of primary graft dysfunction following lung transplantation. Hemopexin (Hx) is the major serum scavenger protein for heme, which is a prooxidant and pro-inflammatory compound. In the current study, we hypothesized that Hx could confer beneficial effects in sterile inflammation induced by IR-mediated lung injury.

**Methods:**

To examine this hypothesis, we administered Hx in an experimental mouse model of unilateral lung IRI.

**Results:**

Our results demonstrate that treatment with Hx alleviated histopathological signs of inflammation in ischemic lungs, as evidenced by a reduction in the number of infiltrating neutrophils and decreased levels of perivascular edema. In addition, thrombotic vaso-occlusion in pulmonary blood vessels of IRI lungs was reduced by Hx. Immunohistochemical analysis revealed that Hx inhibited the up-regulation of heme oxygenase-1, an enzyme highly induced by heme, in ischemic lungs. Finally, Hx administration caused a decrease in the levels of circulating B- and CD8+ T-lymphocytes in the peripheral blood of mice with pulmonary IRI.

**Conclusion:**

These findings suggest that the serum heme scavenger protein Hx holds therapeutic promise in alleviating lung IRI-mediated sterile inflammation. Thus, Hx may represent a preemptive therapeutic approach in IR-related lung disorders such as primary graft dysfunction in lung transplantation.

## Introduction

Pulmonary IRI occurs in various clinical settings including lung transplantation (LuTx), cardiopulmonary bypass surgery, pulmonary embolism and resuscitation for circulatory arrest (1, 2). Notably, in LuTx pulmonary IRI is the main cause of primary graft dysfunction (PGD) and is also associated with a higher risk of chronic lung allograft dysfunction (3–5). Clinical manifestations of lung IRI are characterized by hypoxemia, non-specific alveolar damage and pulmonary edema, all of which are consequences of sterile inflammation. Sterile inflammation in the lung is caused by an innate immune response and encompasses various pathophysiological events such as activation of the pulmonary endothelium and recruitment of neutrophils (6–8). Although a growing number of clinical and experimental studies have enhanced the understanding of the regulatory mechanisms in lung IRI, effective therapies for treating this condition remain elusive [for a recent review see (5)].

Heme, an iron-containing prooxidant and pro-inflammatory compound (9, 10), has been implicated in the pathogenesis of IRI in kidney and heart (11, 12). In particular, ‘free’, i.e. non-protein bound, heme has been shown to be a damage-associated molecular pattern (DAMP) that mediates pro-inflammatory effects via Toll-like receptor (TLR)-4 signaling (13, 14). Hemopexin (Hx), a serum protein with high binding affinity for heme (K_D_ 10^-14^), is the principal scavenger protein of circulating heme. In particular, Hx can protect the endothelium from inflammatory activation and exhibits potential as a therapeutic agent for treating hemolytic disorders such as sickle cell disease (SCD) (14–18). More recently, Hx has been investigated in a clinical multicenter phase 1 trial on SCD patients (NCT04285827) (18, 19).

The primary goal of the current study was to assess the therapeutic potential of Hx for treatment of lung IRI. To this end, we established a preclinical mouse model of unilateral lung IRI that has previously been employed in rats for drug testing of pirfenidone (20). Our findings demonstrate that Hx alleviates pro-inflammatory alterations associated with lung IRI, thereby suggesting its potential value as a preemptive strategy for PGD in LuTx.

## Materials and methods

### Animals

C57BL/6J (B6) male mice were used (Jackson Laboratory, Sacramento, California, 25-30 g/bodyweight, 12-15 weeks of age) throughout the study. Mouse experiments were in accordance with the German Animal Welfare Act and the European directive for animal experiments. The study protocol was approved by the Lower Saxony State Office for Consumer Protection and Food Safety (No. 20-3577).

### Model of unilateral pulmonary IRI in mouse

**Figure 1:**
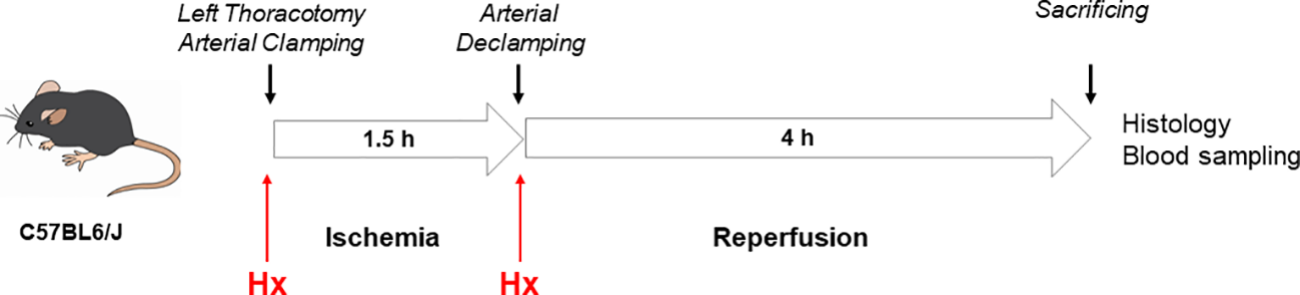
Mouse model of unilateral lung IRI. A model of unilateral pulmonary IRI was established in C57BL6/J mice by unilateral pulmonary clamping of the left lung hilum for 1.5h after left-sided thoracotomy followed by 4 h of reperfusion.

B6 mice were anesthetized with 1% isoflurane (Abbvie Inc. North Chicago, Illinois) and 1L/min oxygen, intubated with a 20G venous catheter and placed under the ventilator (UNO micro-ventilator, UNO Röstvaststaal, Zevenaar, Netherlands; rate 120/min, pressure controlled 12 cm H_2_O, PEEP 2 cm H_2_O). Analgesia was administered subcutaneously with 150 mg/kg body weight metamizole (Ratiopharm GmbH, Ulm, Germany), after which the animal underwent a left-sided thoracotomy. A model of unilateral warm IR of the left lung was established in B6 mice by unilateral pulmonary clamping of the left lung hilum for 1, 1.5 and 2 h after thoracotomy followed by 4 h of reperfusion, as previously demonstrated by others (21). After histological evaluation of these various experimental settings an ischemia time of 1.5 h followed by 4 h of reperfusion was applied for the experiments of this study. Mice were divided into two groups, in which the treatment group received human plasma derived Hx (CSL Behring) (2 x 2 mg: 0.07-0.08 mg/g body weight, n=5) intraperitoneally, before and after lung hilum clamping. The vehicle group of mice was treated with 0.9% sodium chloride (n=5). After sacrificing, blood was taken from the inferior vena cava, lungs were rinsed with heparin-containing (2 IU/ml) phosphate-buffered saline (PBS) and the heart-lung block was removed (**Figure 1**).

### Evaluation of the unilateral pulmonary IRI mouse model

To evaluate the experimental IRI mouse model, the extent of acute lung injury (ALI) was determined according to previous studies (22, 23). Specifically, ten view fields per lung section were assessed with hematoxylin-eosin (HE)-staining (400-fold magnification) of left and right lungs, respectively. The evaluation criteria included congestion, edema, inflammation and hemorrhage. Individual criteria received a score of 0 to 4 and were added for a total score, respectively.

### Histopathology

The removed heart-lung block was left in a 3.7% buffered formalin solution overnight. The lung was sectioned transversely and embedded in paraffin. Paraffin sections were stained at the Institute of Pathology (Hannover Medical School) with HE, Elastica-van-Gieson and periodic acid Schiff (PAS), respectively. Thrombus formation in pulmonary vessels was evaluated in HE stained lung sections by determining the number of thrombi relative to the number of vessels.

### Evaluation of the number of infiltrating neutrophils in lung tissue

Infiltrating neutrophils were counted for quantitative evaluation of pulmonary sterile inflammation. In PAS staining, ten view fields per section of right and left lungs were evaluated at 400-fold magnification by light microscopy, respectively. Infiltrating neutrophils were identified by the eosinophilic granular cytoplasm and the circular nucleus and neutrophils in vessels were excluded.

### Evaluation of perivascular edema

The method for assessing perivascular edema has previously been described (24, 25). After Elastica-van-Gieson staining five vessels in left and right lungs were assessed with a phase contrast microscope (200-fold magnification). Shortest distances from tunica intima to tunica intima were compared with the shortest diameter from tunica adventia to tunica adventia. The shortest distances were considered as true values to compensate for a distortion caused by different cutting planes. To compare different vessel sizes, the results were combined into one parameter using the formula cuff rea/vessel area = D_c_^2^-D_V_^2^/D_V_^2^ (24).

### Immunohistochemistry

Expression levels of HO-1 in the lung were determined by immunohistochemistry, as previously described (11).

### Blood sampling and isolation of immune cells

After reperfusion, mice were sacrificed with whole blood collection. One drop of the blood was suspended in 1 ml PBS containing 2 IU/ml heparin. Peripheral blood mononuclear cells (PBMCs) from blood suspensions were isolated by gradient separation using Ficoll solution (Biocoll Separating Solution; Biochrom GmbH, Berlin, Germany), as indicated by the manufacturer. Isolated cells were kept on ice, stained and used for flow cytometry.

### Flow cytometry

The gating strategy was performed as previously described (26). All antibodies were obtained from Biolegend (San Diego, CA, USA). PBMCs were stained with fluorescently labeled anti-mouse antibodies to CD45 (APC/Cy7, clone 30-F11) and CD19 (APC, clone 6D5) to identify B cells. They were detected in the lymphocyte area in the SSC-FSC window. CD45 (APC/Cy7, clone 30-F11), CD3 (PE/Cy7, clone 17A2), CD4 (FITC, clone RM4-5) and CD8 (PerCP/Cy5.5, clone 53-6.7) were used to identify T cells (CD45^+^CD3^+^CD4^+^CD8^-^ as helper T cells and CD45^+^CD3^+^CD4^-^CD8^+^ as cytotoxic T cells) in the same lymphocyte area. Dendritic cells (DCs) were determined by CD45 (APC, clone 30-F11), CD11b (PE/Cy7, clone M1/70) and CD11c (PE, clone N418) in the whole leukocytes´ area. An Attune™ NxT flow cytometer (Thermo Fisher Scientific, Waltham, Massachusetts, USA) was used. The data were analyzed by FlowJo software (FlowJo X 10.0.7r2, Becton, Dickinson & Company, Franklin Lakes, New Jersey, USA).

### Statistics

Statistics were carried out with Graph Pad Prism 8, Version 8.4.3 (GraphPad Prism Software Inc.). For statistical analysis, each experimental group included five mice. Results are presented as median (interquartile range: IQR). The data were evaluated using the unpaired Mann-Whitney test. A p-value <0.05 was recognized as statistically significant.

## Results

### Hx alleviates sterile inflammation in pulmonary IRI

**Figure 2:**
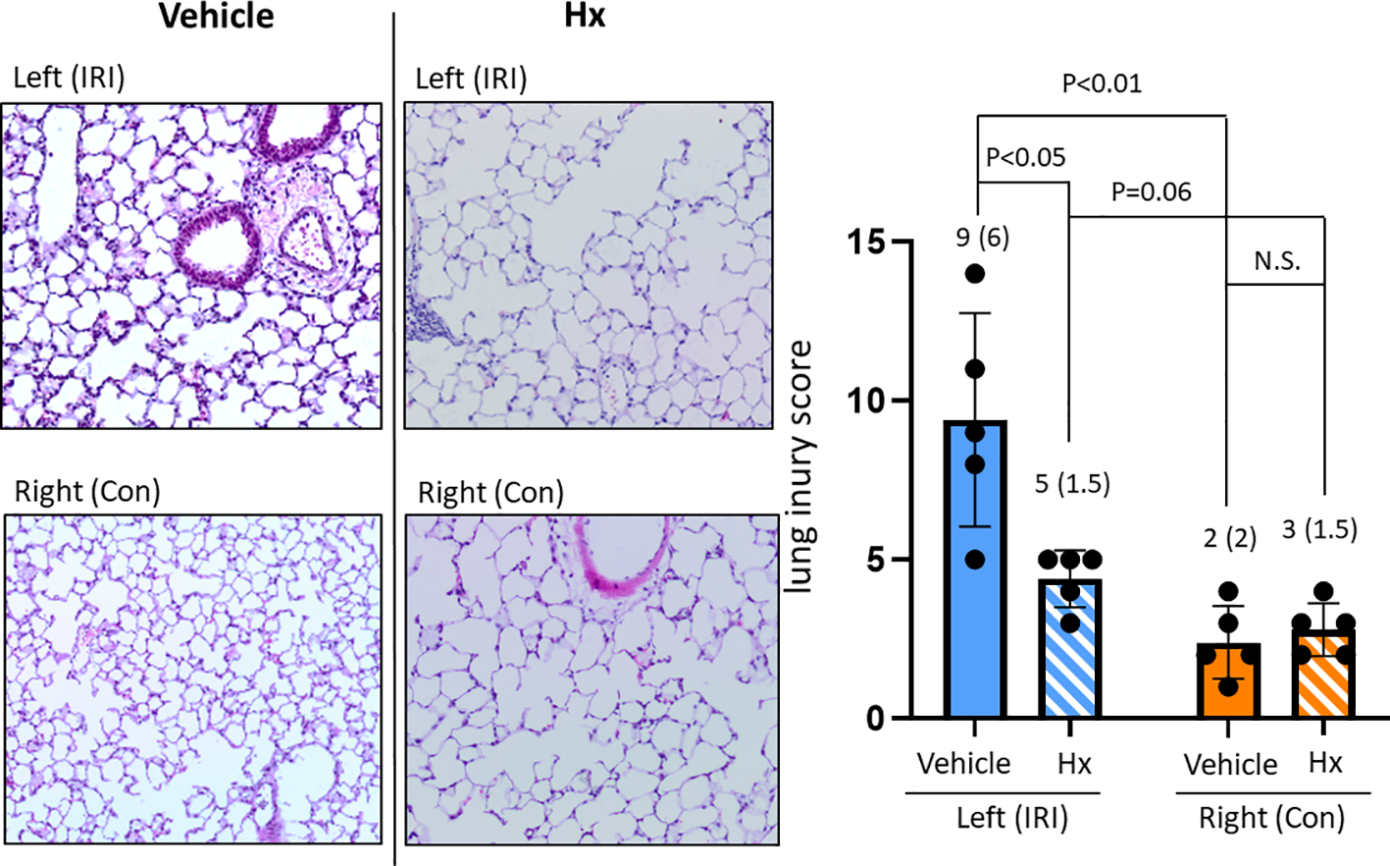
Comparing lung injury scores between ischemic and control lungs in the unilateral pulmonary IRI mouse model - treatment with Hx. Lung tissue damage in the applied mouse model of unilateral pulmonary IRI was evaluated according to the lung injury score by Murakami et al. (22). In this scoring system, lung injury was graded on a scale of 0 to 4 (0, normal; 1, light; 2, moderate; 3, strong; 4 intense) for congestion, edema, infiltration, and hemorrhage in multiple areas (10 microscopic view fields). The left ischemic lung (IRI) exhibited significantly higher levels of lung injury than the right control lung (Con) (IRI vs. Con: 9 (6) vs. 2 (2), respectively: p = 0.02, IRI vs. Sham: 9 (6) vs. 0 (0), respectively: p = 0.008). Moreover, in the group of mice treated with Hx (IRI group vs. Hx treated group: 9 (6) vs. 5 (1.5), respectively: p = 0.037). (n=5 mice in each group). N.S., not significant.

**Figure 3:**
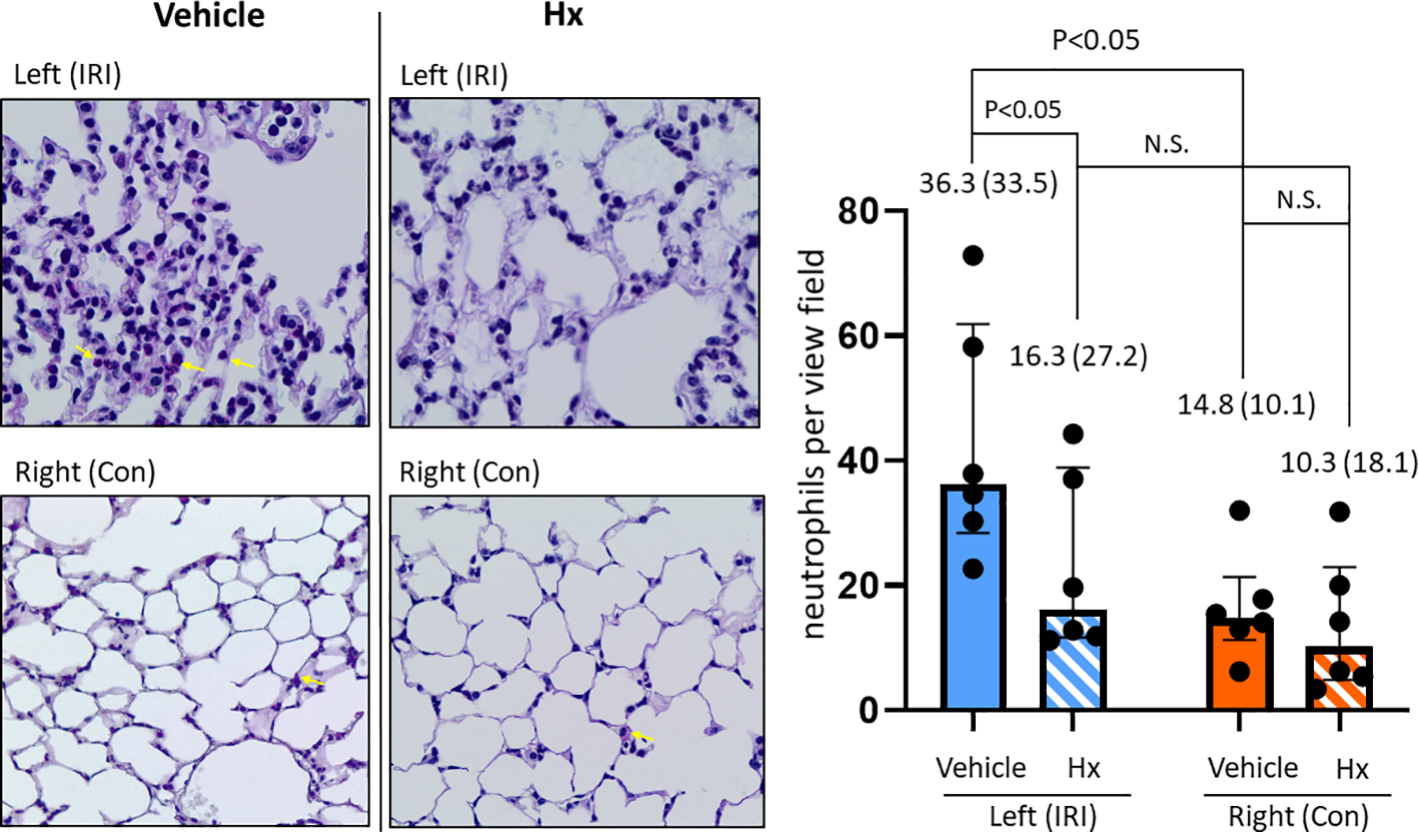
Hx reduces number of infiltrating neutrophils in the ischemic lung of the unilateral pulmonary IRI mouse model. Infiltration of neutrophils was histologically determined by PAS staining, as detailed in Materials and Methods. The indicated red-dyed cells (yellow arrows) are neutrophils. Numbers of neutrophils were significantly higher in the left ischemic compared to the right control lung (36.3 (33.5) vs. 14.8 (10.1), respectively: p = 0.047). In the group of Hx-treated mice significantly lower numbers of neutrophils were determined in the left ischemic lung (IRI group vs. Hx treated group: 36.3 (33.5) vs. 16.3 (27.2), respectively: p = 0.047). n=6 mice for the vehicle group (IRI and Con), and, n=6 mice for the Hx-treated group (IRI+Hx and Con+Hx). N.S., not significant.

In the current study, we used a mouse model of experimental lung IRI induced by unilateral left pulmonary arterial clamping (**Figure 1**). In the treatment group, Hx was administered directly before and after lung arterial clamping (**Figure 1**) and the vehicle group was treated with saline. Ischemic lungs (IRI) exhibited significantly higher levels of lung injury than control lungs (Con) (**Figure 2**). Treatment with Hx markedly reduced the extent of lung injury (**Figure 2**). To evaluate whether pulmonary IRI induces sterile inflammation neutrophils were counted in ischemic and control lungs (5–7). Numbers of infiltrating neutrophils in left ischemic lungs were significantly higher relative to right control lungs (**Figure 3**). However, compared to vehicle-treated mice neutrophil counts were markedly lower in IRI lungs of Hx-treated mice. Notably, Hx had only a minor effect on the number of neutrophils in non-ischemic control lungs (**Figure 3**).

**Figure 4:**
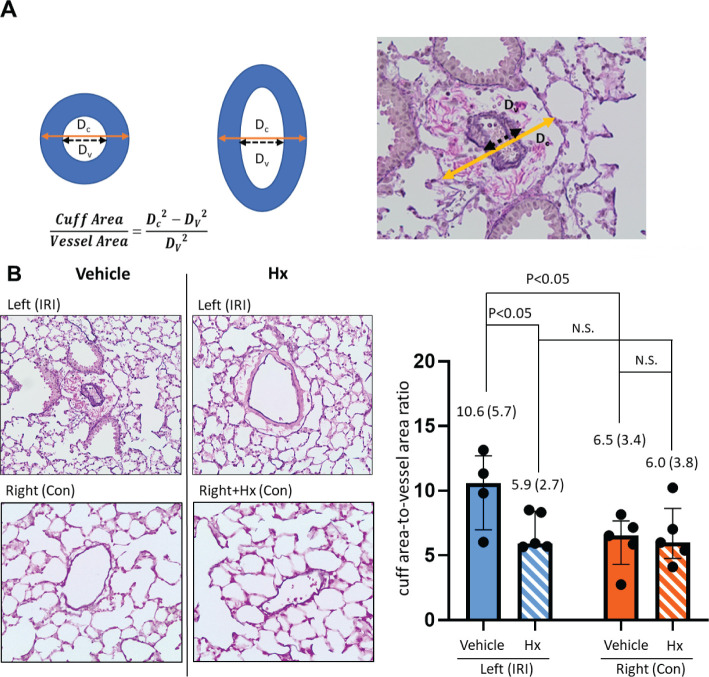
Hx alleviates perivascular edema in ischemic lungs of the unilateral pulmonary IRI mouse model. **(A)** Levels of perivascular edema were evaluated histologically by assessing five vessels of right and left lungs, respectively. Distances from tunica intima to tunica intima (Dv) were compared with the shortest diameter from tunica adventia to tunica adventia (Dc). Shortest distances were utilized as measured values to compensate for distortion of the results caused by different cutting planes. To compare different vessel sizes, the results were combined into one parameter with the formula cuff rea/vessel area = D_c_^2^-D_V_^2^/D_V_^2^, as indicated. **(B)** The extent of perivascular edema, as determined by the cuff area-to-vessel area ratio, was markedly higher in the left ischemic compared to the right control lung (10.6 (5.7) vs. 6.5 3.4), respectively: p = 0.031). Increased levels of cuff area-to-vessel area ratio in ischemic lungs were reduced by treatment with Hx (IRI group vs. Hx treated group: 10.6 (5.7) vs. 5.9 (2.7), respectively: p = 0.032). n=5 mice for the vehicle group (IRI and Con), and, n=5 mice for the Hx-treated group (IRI+Hx and Con+Hx). (Outlier [35.4] in the IRI with vehicle group was excluded). N.S., not significant.

An early indicator of vascular inflammation is the level of perivascular edema that can be measured by quantification of the cuff area-to-vessel area ratio (24, 25) (**Figure 4A**). The comparison of cuff area-to-vessel area ratios in IRI- and control lungs revealed significantly higher levels of perivascular edema in IRI lungs (**Figure 4B**). Notably, in mice treated with Hx perivascular edema in ischemic lungs was markedly reduced. No effect of Hx was observed in control lungs (**Figure 4B**). Overall, these findings indicate that pulmonary IRI induces sterile inflammation in the lung that is alleviated by treatment with Hx.

### Hx alleviates vascular thrombosis in pulmonary IRI

**Figure 5:**
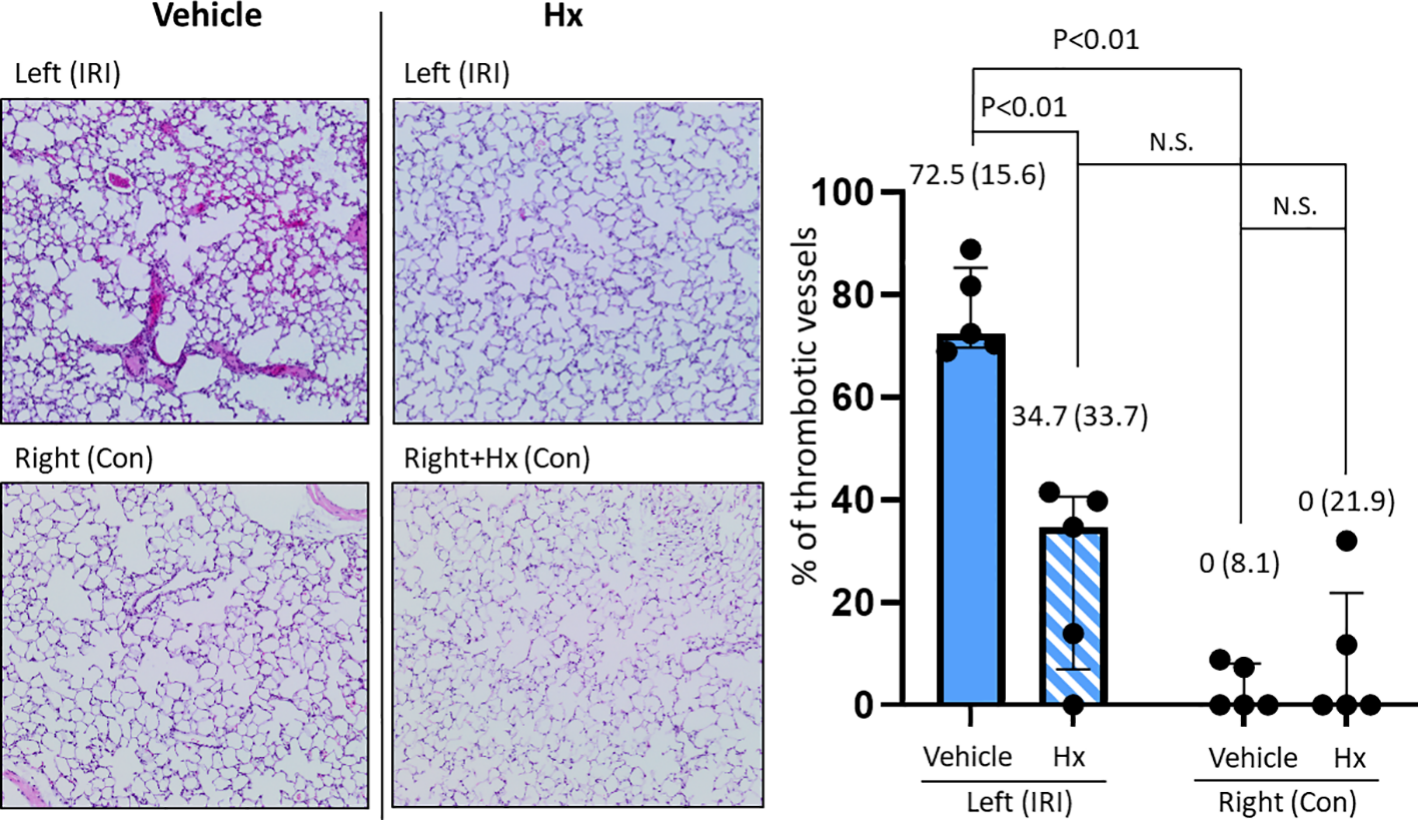
Hx alleviates intravascular thrombus formation in the ischemic lung of the unilateral pulmonary IRI mouse model. Histological studies revealed significantly higher numbers of thrombotic blood vessels in the left ischemic compared to the right control lung (72.5 (15.6)% vs. 0 (8.1)%, respectively: p = 0.008). The number of thrombotic pulmonary vessels was significantly reduced in the group of mice treated with Hx (IRI group vs. Hx treated group: 72.5 (15.6)% vs. 34.7 (33.7)%, respectively: p = 0.008). n=5 mice for each group. N.S., not significant.

Free heme can induce coagulation in blood vessels via activating the vascular endothelium (27). To assess the extent of thrombotic vasoocclusion, we counted thrombotic vessels in both IRI and control murine lungs. Indeed, the number of thrombotic blood vessels was significantly higher in IRI lungs and was reduced in the group of mice treated with Hx relative to the vehicle group. No signs of vasoocclusion were observed in the control lungs after experimental ischemia with or without Hx (**Figure 5**). Thus, pulmonary IRI is associated with vascular occlusion that is alleviated by treatment with Hx.

### Hx inhibits up-regulation of HO-1 expression in pulmonary IRI

**Figure 6:**
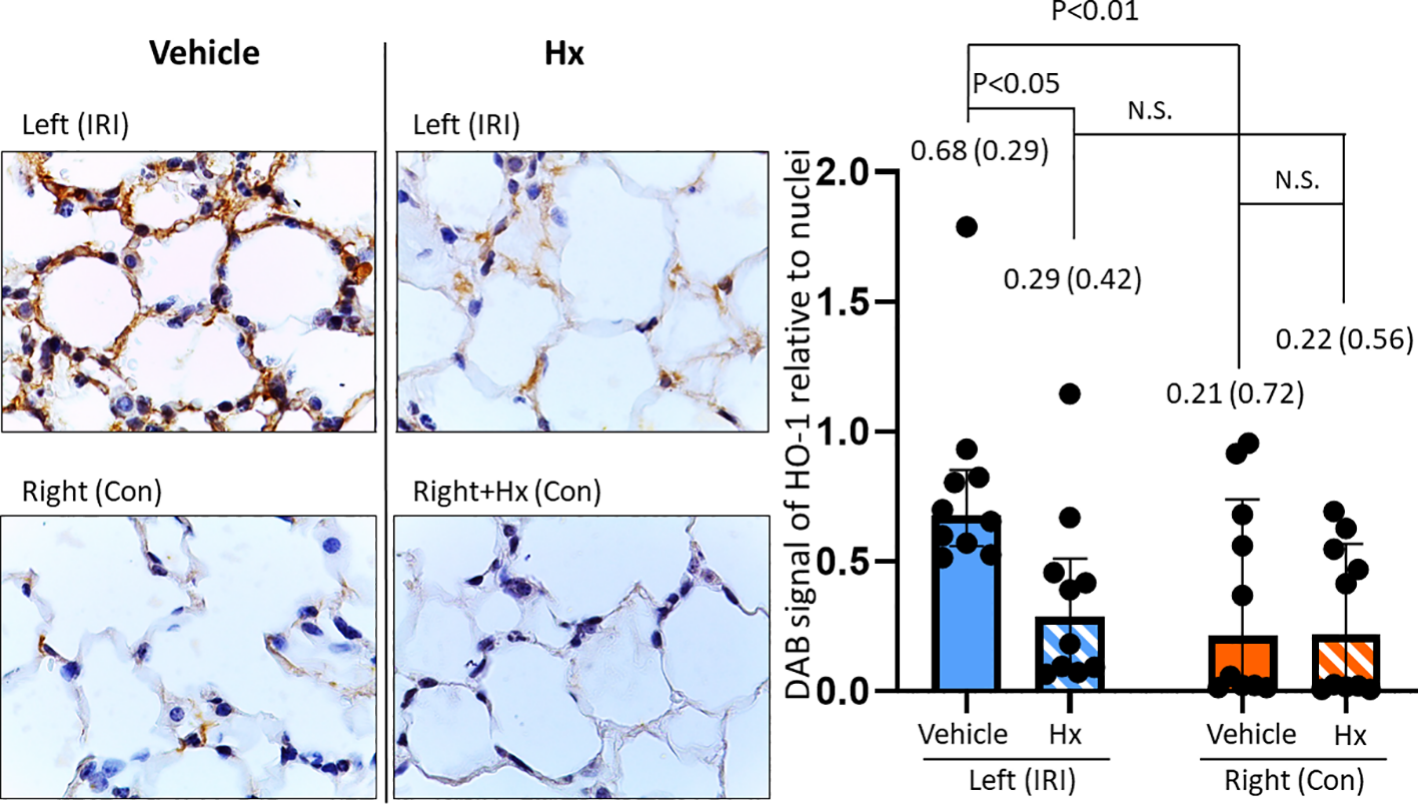
Hx blocks up-regulation of HO-1 expression in the ischemic lung of the unilateral pulmonary IRI mouse model. Immunohistochemical studies revealed significantly higher levels of HO-1 expression in the left ischemic relative to the right control lung (0.68 (0.29) vs. 0.21 (0.72), respectively: p = 0.005). In the group of Hx-treated mice, HO-1 expression levels were significantly reduced in the ischemic left lung (IRI group vs. Hx treated group: 0.68 (0.29) vs. 0.29 (0.42), respectively: p = 0.049). n=10 (5 lungs/mice, two measurements for each lung in each group. N.S., not significant.

Free heme has been implicated in the pathogenesis of IRI-mediated sterile inflammation (11, 12). To further investigate the potential role of heme in IRI-dependent sterile pulmonary inflammation, we assessed the expression of the heme-inducible enzyme HO-1 with immunohistochemical studies. HO-1 protein was up-regulated in the left ischemic lung, but not in right control lungs (**Figure 6**). In mice treated with Hx, HO-1 levels were significantly reduced in the left IRI lung (**Figure 6**). The data suggest that free heme plays a critical role for mediating inflammation in pulmonary IRI.

### Hx modulates levels of circulating immune cells in the peripheral blood

**Figure 7:**
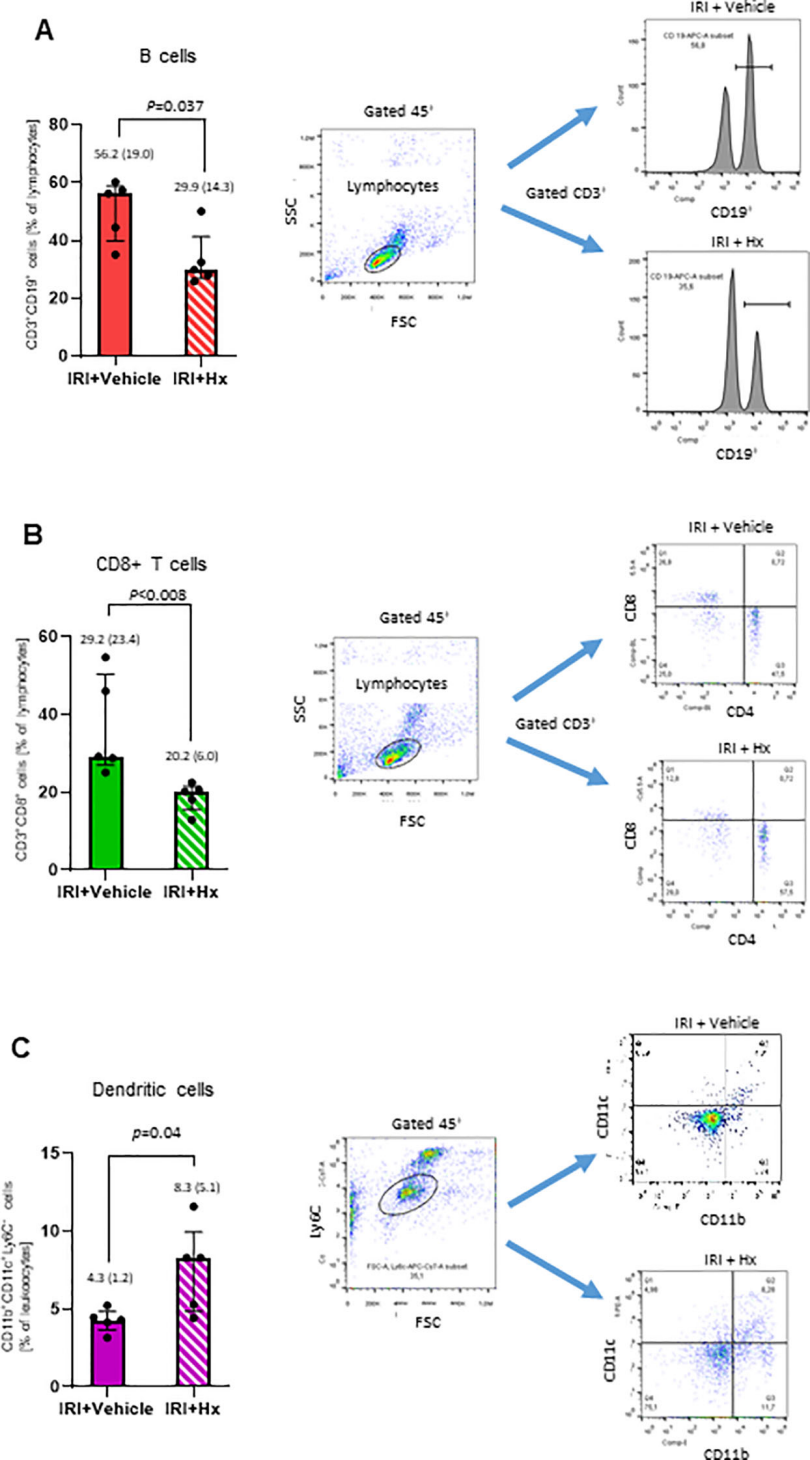
Hx affects levels of immune cells in peripheral blood from mice with unilateral lung IRI. The gating strategy for flow cytometry analysis of various immune cells is described in Materials and Methods. **(A, B)** Significant lower levels of B- (CD3^+^CD19^+^) and CD8+ T (CD3^+^CD8^+^) cells were detected in IRI mice with Hx compared to mice without Hx treatment (n = 5 mice in each group: p=0.037 and 0.008, respectively). **(C)** Significantly higher levels of dendritic cells were found in IRI mice with Hx treatment than mice without Hx treatment (n = 5, p=0.04).

The IRI-mediated innate immune response may be associated with activation of the adaptive immune system. To further probe into the crosstalk between the two immune systems, we analyzed immune cell populations in the peripheral blood of IRI mice with or without Hx treatment. We observed significantly lower levels of circulating B- and CD8+ T-lymphocytes in mice with pulmonary IRI after treatment with Hx (**Figures 7A, B**). Conversely, numbers of DCs were higher in IRI mice after treatment with Hx (**Figure 7C**). Notably, no significant differences were observed for CD4+ T-cells or natural killer cells between Hx-treated and non-treated groups of mice (**Supplementary Figures S1A, B**). In conclusion, administration of Hx regulates levels of adaptive immune system cells in mice with experimental lung IRI.

## Discussion

Pulmonary IRI is a major risk factor for developing PGD in LuTx that is characterized by sterile inflammation (5, 7). In this study, we aimed to explore the therapeutic potential of the serum protein Hx in a preclinical mouse model of unilateral lung IRI. Our findings underscore the anti-inflammatory effects of Hx and highlight its potential as a therapeutic compound for the clinical management of LuTx.

### Mouse model of unilateral lung IRI for investigating preventive treatment with Hx

In the current study, a mouse model of unilateral lung IRI has been established to investigate whether and how preemptive administration of Hx before reperfusion may have beneficial effects in pulmonary IRI (**Figure 1**). IRI-mediated sterile inflammation plays a critical role in LuTx, but currently no effective therapeutic strategies are available to treat this condition (5). We adapted a rat model of unilateral pulmonary IRI to B6 mice (20). To evaluate the severity of IR-mediated lung injury in this mouse model, we utilized two different scoring systems. According to the lung injury scoring method proposed by Murakami and colleagues (22, 23) we observed significantly worse histopathological scores for pulmonary injury in IRI compared to control lungs (**Figure 2**). In contrast, the second scoring system from the American Thoracic Society (28) revealed less pronounced differences (data not shown). Consequently, we applied the first scoring method throughout this study.

### Hx alleviates sterile inflammation in lung IRI

IRI is linked with a cascade of pathophysiological events that trigger sterile inflammation (6). In various solid organs, mechanisms of IRI are characterized by distinct tissue-specific and context-dependent alterations. However, common principles such as activation of the endothelium, generation of reactive oxygen species (ROS) from mitochondrial dysfunction or other redox-dependent regulatory events are similar in various solid organs (29, 30). Our current report demonstrates that administration of Hx alleviates sterile inflammation in a preclinical mouse model of lung IRI. Specifically, Hx reduces recruitment of inflammatory cells to the lung, decreases endothelial cell activation and lowers the number of thrombotic pulmonary vessels (**Figures 3**–**5**). Free heme, a DAMP that induces TLR4 signaling, has been shown to cause endothelial activation in various experimental models (27, 31). Thus, our current findings are consistent with these reports. Notably, the increased cuff-area-to-vessel ratios observed in IRI lungs (**Figure 4**), an early indicator of vascular endothelial inflammation, indicate that free heme-dependent activation of the endothelium may be critical in driving sterile pulmonary inflammation that can be mitigated by Hx. In addition, pro-inflammatory effects of heme in lung IRI exacerbated by high-mobility group box 1 (HMGB1), a molecule that is known to significantly contribute to the pathogenesis of IRI in various solid organs (32). The major pathogenic role of free heme in lung IRI is also consistent with the observed expression pattern of HO-1, a heme-inducible enzyme (**Figure 6**). The elevated levels of HO-1 in IRI-lungs are markedly reduced in Hx-treated mice (**Figure 6**), likely due to neutralization of free heme by Hx. It remains unclear whether the observed vascular thrombosis in IRI lungs (**Figure 5**) is directly caused by free heme-dependent complement activation (33) or by indirect heme-dependent endothelial cell activation leading to subsequent coagulation. Finally, the specific source of free heme in the applied model of lung IRI is not clear. Free heme could potentially originate from hemoproteins released by necrotic cells during IRI (34) or from damaged red blood cells infiltrating the lung tissue in the reperfusion phase (5, 12). To address this question, we are currently conducting experiments to quantify local levels of free heme in lung IRI using an approach previously applied for kidney IRI (11).

### Lung IRI affects the adaptive immune system

Our findings also reveal a dynamic regulation of various cell populations with the adaptive immune system in peripheral blood following lung IRI (**Figure 7**). For example, DCs play a multifaceted role in the lung, including antigen presentation, immune activation, tolerance induction, and inflammation resolution. DCs serve as an orchestrator of both, innate and adaptive immune responses, within the pulmonary microenvironment (35). Specifically, pulmonary DCs contribute to immune tolerance and homeostasis by promoting the development of regulatory T cells (Tregs). They also aid in resoluting inflammation by the production of anti-inflammatory cytokines that facilitate apoptosis of activated T cells (36). The observed effect of Hx on the regulation of circulating B- and T-cells, both of which play critical roles in immune responses in solid organ transplantation (37) support a crosstalk between the innate and adaptive immune systems during the early phase of IRI. However, much remains to be elucidated to comprehensively grasp the intricate details of how these two immune systems interact and influence each other in the setting of lung IRI (32).

### Translational potential of Hx as a therapy of lung IRI in the clinic

Currently, there are no therapies available to prevent the detrimental consequences of lung IRI in clinical settings such as primary PGD in LuTx (5, 38). Therefore, preemptive administration of Hx before reperfusion in lung IRI could be a promising therapeutic approach to mitigate sterile inflammation and its harmful effects during donor organ procurement for LuTx. Additionally, other serum heme-binding proteins such as α1-antitrypsin and α1-microglobulin also exhibit protective effects by binding heme (39, 40) and may offer complementary benefits in this context. Clinical trials are currently evaluating the therapeutic efficacy of Hx in patients with SCD (19) and will help to identify potential adverse effects, including renal complications, as previously reported (41–43). Furthermore, administration of Hx could contribute to a comprehensive multi-agent therapeutic strategy in IR-mediated lung injury, similar to strategies proposed for SCD (44). In such a multimodal approach, Hx could be combined with other pharmacological approaches such as targeting pulmonary NADPH oxidases (45, 46), adenosine A1 receptors (47) or TLR4 signaling (48).

In conclusion, the present findings suggest that the serum heme-scavenger protein Hx holds major promise as a preventive therapy for IR-related lung damage. To further explore the clinical potential of Hx in LuTx, we plan to investigate its effects in a mouse model of LuTx following the approach previously used for the acute phase serum protein α1-antitrypsin (26). Moreover, further clinical trials are warranted to assess the efficacy and safety of Hx treatment for lung IRI. Such trials could provide valuable insights into the potential benefits of Hx therapy and its role in optimizing clinical outcomes in LuTx.

## Data Availability

The original contributions presented in the study are included in the article/**Supplementary Material**. Further inquiries can be directed to the corresponding author.

## Supplementary material

Supplementary Figure 1 No significant differences in helper T-cells or natural killer cells after Hx treatment. No significant differences were observed for CD4+ T-cells **(A)** and natural killer cells **(B)** in Hx- and vehicle-treated IRI lungs (n = 5 mice for each group, both p = 0.15).
