# Supplementary figures and images for: Hemopexin alleviates sterile inflammation in ischemia-reperfusion-induced lung injury
Source: Front Immunol. 2024 Oct 4;15:1451577. doi: 10.3389/fimmu.2024.1451577 (PMC11487521; doi:10.3389/fimmu.2024.1451577)

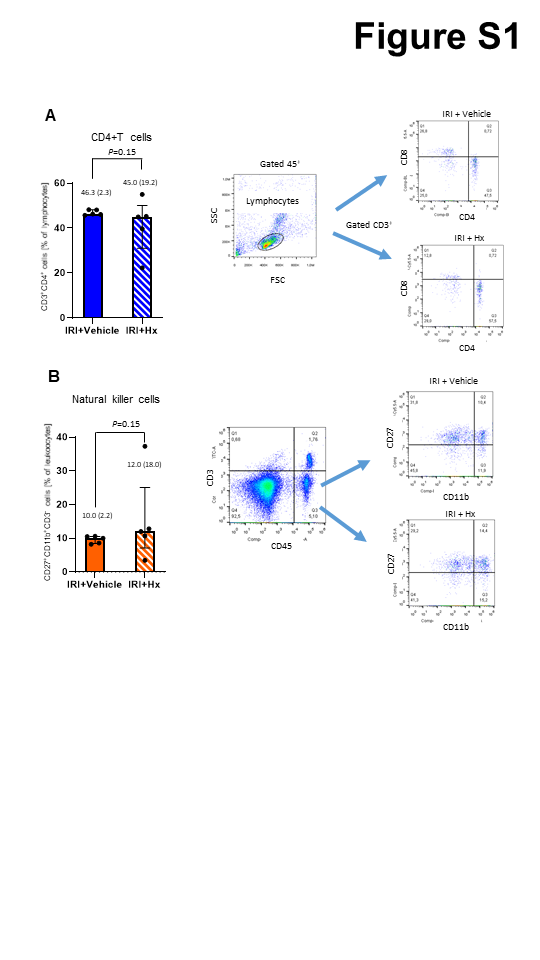

Supplement: Supplementary Figure 1 — No significant differences in helper T-cells or natural killer cells after Hx treatment. No significant differences were observed for CD4+ T-cells (A) and natural killer cells (B) in Hx- and vehicle-treated IRI lungs (n = 5 mice for each group, both p = 0.15). [file Image1.tif]
